# Pathogenicity of Rift Valley fever virus: Organ-specific clinical outcomes and hidden drivers of virulence - a narrative review

**DOI:** 10.1080/21505594.2026.2655047

**Published:** 2026-04-02

**Authors:** Fiona Nicole, Anne-Flore Legrand, Marie-Pierre Confort, Maxime Ratinier, Cyrille Mathieu, Pierre-Yves Lozach, Frédérick Arnaud

**Affiliations:** a IVPC UMR754, INRAE, Universite Claude Bernard Lyon 1, EPHE, Université PSL, Lyon, France; b CIRI, Centre International de Recherche en Infectiologie, Team NeuroInvasion, Tropism and Viral Encephalitis, Univ Lyon, Inserm, U1111, CNRS, UMR5308, Université Claude Bernard Lyon 1, Ecole Normale Supérieure de Lyon, Lyon, France

**Keywords:** Rift Valley fever virus, pathogenesis, virulence, field strains, reassortment, infection routes, liver, brain, placenta, retina, organotypic models

## Abstract

Rift Valley fever virus (RVFV) is a mosquito-borne phlebovirus posing major threats to human and animal health. Despite decades of research, the determinants governing diverse clinical manifestations remain incompletely understood. This narrative review examines four central facets: i) exposure route impacts on disease outcome; ii) field strain diversity and organ-specific virulence; iii) genome reassortment driving viral evolution; and iv) organ-specific pathology including the overlooked placental/retinal infections. Differences among infection routes, viral strains, and host species complicate extrapolation from experimental models to outbreaks. Collectively, evidence indicates that RVFV pathogenesis results from interplay between infection route, viral determinants, and host factors including age and immune responses, which shape viral dissemination, tissue tropism, and organ-specific disease outcomes. Despite progress, major uncertainties remain regarding mechanisms controlling tissue tropism and field strain diversity roles in severe manifestations. Integrating field isolates, organotypic cultures, and physiologically relevant models will be essential to refine understanding and improve prevention.

## Introduction: An old virus with modern questions

**Figure 1.**
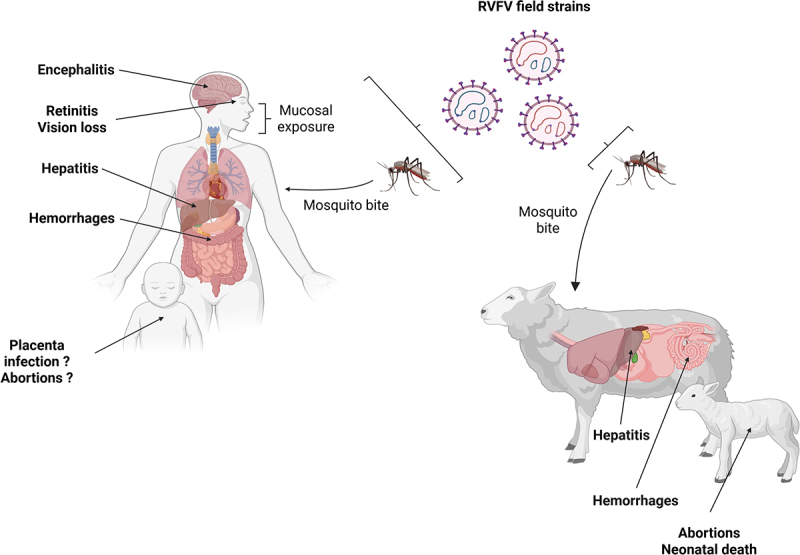
Mechanisms of Rift Valley fever virus (RVFV) transmission and associated disease manifestations in humans and livestock. RVFV is predominantly transmitted to livestock through the bite of infected mosquitoes during hematophagy, while human infection also occurs via mucosal exposure to contaminated tissues or bodily fluids. Vertical transmission in livestock drives abortion storms and elevated neonatal mortality, whereas such outcomes remain insufficiently documented in humans. While RVFV infection is asymptomatic in the majority of human cases, it can cause severe disease characterized by acute hepatitis, hemorrhagic complications, encephalitis, as well as ocular disease and, in the most critical cases, multiorgan failure. In livestock, RVFV similarly induces hepatic injury and hemorrhagic pathology, with young animals being particularly susceptible to severe outcomes. Created with BioRender.com.

RVFV (genus *Phlebovirus*, family *Phenuiviridae*) is an enveloped, negative-sense RNA virus with a tripartite genome (L, M, S) [1]. Since its first isolation in Kenya in 1930, RVFV has caused recurrent epidemics across sub-Saharan Africa and the Arabian Peninsula, characterized by catastrophic livestock losses (abortion storms and high neonatal mortality) and severe human disease manifestations, including hepatitis often accompanied by hemorrhagic complications, as well as encephalitis and ocular infection (retinitis), with reported human mortality rates ranging from 1% to 3% [2]. Transmission is classically mosquito-borne (notably by *Aedes* and *Culex* species), but mucosal contact with infected tissues or fluids is also a well-established route, particularly among farmers, abattoir workers, and veterinarians [3]. Climate change, land-use shifts, and livestock trade are expanding the ecological range of RVFV, making current knowledge gaps and control strategies increasingly critical [4,5]. Several recent reviews have synthesized current knowledge [6–12], notably on RVFV pathogenesis, yet important uncertainties remain. This review highlights recent advances and identifies major knowledge gaps, particularly, in understudied areas of RVFV pathogenesis. Nearly a century after its discovery, key aspects of RVFV pathogenesis remain unresolved. In both ruminants and humans, RVFV causes multi-organ disease, manifesting as severe hepatic injury and abortion storms in ruminants, as well as encephalitis and retinitis in humans (Figure 1) [11,13].

Key unresolved questions include: (i) how different routes of exposure (mosquito bite *versus* mucosal contact) influence disease trajectories; (ii) how genetic diversity among field strains shapes virulence, tissue tropism, and transmission; (iii) the frequency and impact of reassortment (genomic segment exchange) on RVFV biology; and (iv) the mechanisms driving organ-specific pathogenesis, particularly in the liver (the primary replication site) and the brain (associated with late-encephalitic outcomes), as well as understudied manifestations such as placental infection and ocular disease, leading to abortions and vision loss, respectively.

This review focuses on four major aspects of RVFV biology: the role of exposure routes, the contribution of viral strain diversity, the impact of genome reassortment, and the mechanisms underlying organ-specific pathogenesis. It synthesizes experimental, clinical, and epidemiological studies addressing RVFV pathogenesis. Relevant literature was identified through searches in PubMed using combinations of the keywords “Rift Valley fever virus,” “pathogenesis,” “virulence,” “neuroinvasion,” and “reassortment.” Priority was given to primary studies providing mechanistic insights into viral determinants, host responses, and organ-specific pathology. Particular attention was paid to recent primary studies while avoiding redundancy with recent reviews, with the aim of highlighting emerging concepts and remaining knowledge gaps in RVFV pathogenesis.

## Route of exposure: A key determinant of RVFV tropism and virulence

The initial phase of an RVFV outbreak is typically marked by waves of abortions among ruminants, often following infection of animals through mosquito bites in rural areas. Early human cases most often arise from mucosal exposure to infected animal tissues and bodily fluids, particularly during farming, slaughtering, or veterinary procedures, as well as from the consumption of contaminated meat or milk [14,15]. Human infection also occurs through bites from infected mosquitoes [16]. Moreover, while occupational exposure to contaminated animal materials represents a major transmission risk, aerosolized particles generated during high-pressure cleaning or animal processing may also contribute to infection, albeit likely with lower frequency.

Several *in vivo* experimental models of RVFV, described in two review articles [9,17], offer a complementary and versatile platform for dissecting pathogenic mechanisms, with each species or mouse strain recapitulating distinct aspects of the disease. BALB/c mice reproduce severe hepatic and neurological outcomes closely mirroring those observed in humans, whereas C57BL/6 and 129 Sv strains more commonly develop early hepatotropic disease, making them well suited for evaluating virulence, tissue injury, and host response. Rodent diversity further enhances model granularity: Wistar and Brown Norway rats typically develop fulminant hepatitis; Lewis and ACI strains show a propensity for encephalitic progression; and hamsters develop acute, severe hepatitis with hemorrhagic features that can rapidly progress to multi-organ failure and high mortality.

At the upper end of translational relevance, non-human primates, most notably the common marmoset, represent the closest clinical counterpart to human RVFV infection, with disease phenotype ranging from hepatic to hemorrhagic or encephalitic presentations depending on the inoculation route. Across laboratories, past and ongoing studies have used a range of infection routes including intraperitoneal, subcutaneous, intranasal, footpad, and aerosol exposure, reflecting varied experimental goals and model-specific endpoints. Nevertheless, only a limited number of investigations have directly compared or clearly differentiated vector-borne, mucosal, and aerosol transmission routes under controlled animal study conditions. Discriminating between the various routes of viral entry remains crucial, as each may differentially shape viral dissemination, organ tropism, and overall disease outcome.

A study in cattle using a strain from the 2006–2007 outbreak compared intranasal, intradermal, and combined inoculation routes. All routes produced viremia, but intranasal exposure resulted in the highest and most sustained levels, together with more severe clinical signs and broader tissue dissemination, despite the relatively high inoculum used (10^7^ PFU) [18]. Yet, in the absence of data on the minimum infectious dose in cattle, it remains difficult to assess how this high inoculum may have influenced the outcome.

Subcutaneous injection, although commonly used in experimental studies, does not accurately replicate natural mosquito transmission, which occurs intradermally and involves additional biological factors such as mosquito saliva and insect-derived viral particles. Moreover, the volume delivered by a mosquito bite differs markedly from that of an injection, and the precise infectious dose introduced during mosquito blood feeding remains poorly defined. In C57BL/6 mice, the ZH548 strain induced primarily hepatic pathology after intraperitoneal inoculation, while intradermal infection, which more closely reflects mosquito-borne transmission, resulted in both hepatic and neurological disease [19]. Notably, co-inoculation with mosquito saliva or salivary gland extracts further increased mortality and elevated viral titers in blood and organs compared to infection alone [19]. Thus, integrating mosquito-derived factors and natural inoculation routes is essential to capture more realistic infection dynamics.

Interestingly, in mice, RVFV pathogenesis also differs between subcutaneous inoculation and aerosol exposure: while both routes yield comparable survival rates and can induce acute hepatitis, aerosol exposure is associated with earlier onset and more severe neuropathology [20]. Moreover, intranasal infection of mice with attenuated strains (Smithburn, Clone 13) resulted in higher mortality and greater brain viral loads than subcutaneous infection, underscoring the importance of the exposure route in determining neurovirulence, at least for attenuated viruses [21]. Overall, these findings suggest that aerosol and intranasal exposure may bypass, or at least accelerate, biological bottlenecks that normally delay neuroinvasion, thereby altering both the pace of disease progression and the range of tissues affected. For veterinary workers, farmers, slaughterhouse staff, and laboratory personnel, the potential for RVFV transmission via mucosal routes or, in specific circumstances, through aerosol exposure constitutes a significant occupational risk. This underscores the need for stringent mucosal and respiratory protection not only during field outbreaks, but also during routine manipulation of infectious material in BSL-3 facilities. From a biodefense perspective, this atypical transmission route, uncommon among arboviruses, warrants particular vigilance, as efficient mucosal entry coupled with accelerated neuroinvasion potential raises notable dual-use concerns, in addition to posing a concrete economic threat to agricultural and livestock sectors.

Taken together, available studies indicate that the route of exposure strongly influences early viral dissemination and disease outcome. Peripheral infection typically results in primary hepatic replication, whereas mucosal or aerosol exposure may facilitate earlier neuroinvasion. However, the precise mechanisms controlling these differences remain incompletely understood. Experimental models that incorporate natural transmission factors such as intradermal inoculation with mosquito saliva, direct mosquito feeding, or mucosal exposure, *e.g*., intranasal route, provide the most physiologically relevant insights. Moreover, infection doses vary widely across studies, and systematic comparisons of high and low inocula could yield valuable information on dose-dependent effects on viral dissemination, pathogenesis, and host responses. Emerging evidence further indicates that sex-related differences and the cellular origin of the virus, reflecting differences in virus production in mammalian *versus* insect cells, can significantly influence infection outcomes [22]. Hence, multiple parameters affect viral tropism, replication kinetics, immune activation, and tissue-specific pathology, underscoring the need for standardized experimental conditions and integrated analytical approaches. Future studies should therefore move beyond convenience routes (*e.g*., intraperitoneal, subcutaneous, footpad, etc.) and systematically account for infectious dose and viral cell origin to more faithfully reproduce the dynamics of natural infection and host responses.

## Field strain diversity: A neglected source of virulence variability

Viruses, including RVFV, display significant phenotypic heterogeneity, even though overall genetic diversity across RVFV genetic lineages is relatively low (between 1 and 5%) [23]. Pioneering work in the 1980s revealed that RVFV strains exhibit marked differences in virulence in rodent models. Indeed, in a comparison of 22 isolates, Battles *et al*. found median lethal doses (LD_5__0_) in outbred mice spanning 2 PFU to over 10^6^ PFU, with an even wider range in C57BL/6 mice [24]. Another study further showed that Egyptian isolates were more virulent in Wistar-Furth rats than sub-Saharan strains [25]. Subsequent studies confirmed that RVFV strains from distinct genetic lineages exhibit heterogeneous virulence in outbred mice. For example, isolates such as SA51 (lineage G), Entebbe (lineage D), and OS7 (lineage E) induced more rapid hepatic injury and severe acute disease than other strains [26]. Chabert *et al*. recently compared two Mauritanian isolates, MRU25010-30 (isolated from a camel, 2010) and MRU2687-3 (isolated from a goat, 2013) [27], belonging to lineages E and A, respectively, as defined in the nomenclature of Bird *et al*. [23]. They reported distinct lethality patterns in BALB/c mice: the former induced rapid death accompanied by high hepatic viral loads and pronounced viremia, whereas the latter, similar to the reference strain ZH548, resulted in delayed death associated with higher viral loads in the brain [27]. Overall, these observations indicate that strain-specific variation contributes to differences in virulence and organ tropism, highlighting the biological importance of viral genetic diversity in shaping RVFV pathogenesis.

Although the precise viral determinants remain unclear, variation in viral entry efficiency, replication kinetics, or immune evasion may influence whether infection results primarily in hepatic disease or delayed neurological involvement. Importantly, the viral population within each strain should also be considered, as this intra-strain diversity (comprising both major and minor variants) may contribute to the global virulence of the virus. Overall, such variation complicates the extrapolation of findings from reference strains extensively used in laboratory settings, such as ZH548 or ZH501, to real outbreak contexts. For translational applications, including diagnostics, antivirals, and vaccine development, it is therefore essential to perform comparative phenotyping of circulating field isolates under standardized protocols. This requires not only expanding the collection of field isolates but also ensuring their accessibility to the broader scientific community. Classifying strains by organ tropism, *e.g*., liver *vs* brain, will help improve our understanding of virulence patterns and, in turn, guide outbreak management and therapy design. In parallel, deeper characterization of field isolates using deep sequencing and reverse genetics approaches will be crucial to uncover the viral determinants underlying severe or divergent disease outcomes.

## Reassortment: A hidden driver of RVFV evolution

Reassortment is a hallmark of *Bunyaviricetes* [28], including RVFV, whose genome is segmented into three RNA segments (L, M, and S). Co-infection of a single host cell by different strains has been shown to enable segment exchange, giving rise to reassortant viruses with novel genetic combinations. A good example is Ngari virus, a recognized cause of hemorrhagic fever in Africa, which emerged from the reassortment of genetic segments between Bunyamwera virus and an unidentified orthobunyavirus [29]. Early genetic evidence from Africa has also shown that natural RVFV reassortment occurs in the field, including between wild-type lineages and vaccine-derived strains, highlighting the need to anticipate potential risks in control strategies [30]. More recently, experimental studies have confirmed the efficiency of reassortment both in vectors and in mammalian hosts. In mosquitoes, co-infection of *Culex tarsalis* with different RVFV strains led to frequent reassortment, highlighting the vector’s potential role as a mixing vessel for viral evolution [31]. In sheep, reassortment was also observed following experimental co-infection, indicating that livestock, the main amplifying hosts in natural outbreaks, can serve as additional platforms for genome reshuffling [32]. Mechanistic insights into these processes highlight the molecular plasticity of RVFV and its capacity to generate novel phenotypes, raising concerns over unpredictable consequences for transmission dynamics and virulence [33]. This adaptability also heightens the prospect of barrier crossing, both across mammalian hosts and between vector species, potentially reshaping ecological range, host susceptibility, and outbreak severity.

Collectively, these findings establish reassortment as a major driver of RVFV evolution and underscore its relevance for surveillance and risk assessment, especially since the phenotypic traits of resulting viruses, including their transmissibility and pathogenicity, remain difficult to predict with our current knowledge. Future research should prioritize systematic surveillance and experimental characterization of RVFV reassortants, together with comprehensive genomic and phenotypic profiling of circulating field strains, to better anticipate their impact on transmission, virulence, and potential risks for control strategies.

## RVFV pathogenesis at the organ level: Liver, brain, and hemorrhagic outcomes

As previously mentioned, RVFV infection causes severe disease in both humans and ruminants: in humans, fulminant hepatitis with hemorrhagic manifestations and delayed-onset encephalitis are characteristic manifestations of severe cases, while in ruminants the disease is marked by massive hepatocellular necrosis with associated hemorrhages [9]. However, the mechanisms underlying these pathologies remain poorly understood.

### Viral determinants modulating organ tropism

In theory, all RVFV proteins may contribute to virulence and disease outcomes, yet only a subset has been clearly demonstrated to directly modulate pathogenicity. For instance, the M segment, which encodes the viral surface glycoproteins Gn and Gc, exhibits the greatest genetic variability within the RVFV genome [23]. Hence, structural differences in these envelope glycoproteins are likely to modulate entry routes, cellular tropism, replication dynamics, and viral morphogenesis [10,34], ultimately shaping whether disease presents with hepatic or neurological manifestations; however, experimental evidence supporting this relationship is not yet available.

Conversely, the NSs protein encoded by the S segment is one of the most extensively characterized RVFV factors, functioning as a major virulence determinant in mammalian hosts [6,10,35]. The contribution of NSs to pathogenicity is primarily attributed to its potent antagonism of type I interferon (IFN-I) signaling, achieved through TFIIH-p62 degradation, suppression of IFN-β promoter activation, and proteasome-dependent inhibition of PKR [6,10,36,37]. NSs is well known to assemble into large nuclear filaments [38], often extending tens of micrometers. These structures were recently shown to consist of bundled ~10-nm amyloid-type fibrils [39]. NSs also forms fibrils in the cytosol, which appear as more amorphous aggregates by fluorescence microscopy, in contrast to the prominent nuclear filaments [39].

These filamentous assemblies are critical to NSs function, particularly in suppressing innate immune signaling. Experimental evidence shows that they not only promote RVFV replication and systemic disease following intraperitoneal infection, but also exacerbate virus-induced neuropathology after intracranial inoculation in mice [39,40]. In mice, subcutaneous or intraperitoneal inoculation with an attenuated ZH501 RVFV strain lacking NSs leads to efficient viral clearance and does not produce a lethal outcome, whereas intranasal infection results in a fatal neuroinvasive disease characterized by high viral load and pronounced central nervous system (CNS) inflammation [39,41]. These findings indicate that a functional NSs protein is critical for efficient hepatic infection, systemic spread, and subsequent progression to CNS following peripheral exposure. Moreover, the attenuated Clone 13 strain, which contains a large deletion in the NSs gene and is non-lethal following subcutaneous inoculation, still produces fatal encephalitis in mice after intranasal administration [21]. Hence, although NSs can facilitate neuropathogenesis, it is not strictly necessary for neuroinvasion and neurological disease *via* the intranasal route, indicating that CNS pathology can arise from robust viral replication within neural tissues independently of NSs.

In addition to NSs, the NSm protein encoded by the M segment also contributes to RVFV virulence in immunocompetent mice [42,43]. Notably, 100% of rats intraperitoneally inoculated with RVFV-ZH501 develop fatal hepatic disease, whereas only 61% of those infected with an NSm-deficient strain succumb, with survivors exhibiting either hepatic (44%) or neurological (17%) manifestations [42]. These findings support a role for NSm in both virulence and clinical outcome modulation in mammalian hosts. NSm also exhibits *in vitro* anti-apoptotic activity mediated by its C-terminal domain, which contains a basic amino-acid cluster and a predicted transmembrane region that localizes the protein to the mitochondrial outer membrane [44,45]; however, the relevance of this function to *in vivo* pathogenicity remains unresolved. Finally, elevated P78 expression has been shown to attenuate RVFV virulence by limiting viral replication in macrophages, indicating that P78 also contributes to modulation of pathogenicity [46]. Hence, NSs likely does not solely explain the full spectrum of organotropism and disease kinetics observed among field strains, as NSm, P78, and potentially additional components such as Gn-Gc, as well as the viral polymerase about which no functional data are currently available, may also modulate virulence and clinical outcome. Further investigation of viral determinants in natural isolates will be essential to delineate the respective contributions of RVFV proteins to pathogenicity.

### Host determinants of hepatic disease: The central role of LRP1

Cell entry is the initial and essential step in viral infection. In RVFV, the envelope glycoproteins Gn and Gc encoded by M segment mediate entry and play a pivotal role in defining cellular, tissue and species tropism in mammalian hosts. To date, two mammalian attachment factors have been identified: the C-type lectin L-SIGN, which is predominantly expressed on liver sinusoidal endothelial and lymph node cells [47], and the broadly distributed heparan sulfate proteoglycans found on virtually all eukaryotic cell surfaces [48,49]. In contrast, the C-type lectin DC-SIGN, highly expressed on dermal dendritic cells, functions as a true endocytic receptor for several phenuiviruses [50]. DC-SIGN is of particular interest because it is expressed on cells situated at the anatomical site of viral transmission by infected mosquitoes.

The identification of an additional host determinant that governs RVFV tropism and pathogenic outcomes has recently advanced our understanding of the molecular basis of liver injury during infection. Indeed, recent studies have identified LRP1 (low-density lipoprotein receptor-related protein 1) as a cellular receptor for RVFV, establishing it as a key determinant of lethal hepatic disease [51,52]. Silencing LRP1 in hepatocytes restricts RVFV ZH501 strain replication, markedly reducing liver damage and redirecting pathogenesis toward the brain in footpad-infected mice resulting in delayed neurological manifestations rather than early fulminant hepatitis [52]. Interestingly, no difference in survival was observed between control mice and those lacking LRP1 in hepatocytes following intranasal infection, indicating that neuroinvasion *via* this route proceeds independently of hepatic LRP1 expression. More recently, the interaction between LRP1 and several bunyaviruses, including RVFV, was shown to be strongly modulated by cellular factors such as the amount of cholesterol and the level of endocytic activity, underscoring a relationship far more complex than a simple virus-receptor interaction [53].

Beyond this key host determinant of RVFV liver infection and disease, studies in lambs (a natural host highly susceptible to RVFV) further show that infection profoundly disrupts hepatic immune and metabolic responses [54]. Transcriptomic analyses showed upregulation of genes involved in immune activation and programmed cell death regulation. Genes encoding metabolic enzymes, including those acting on lipids, carbohydrates, amino acids, nucleic acids, and vitamins, were also upregulated. Conversely, xenobiotic metabolism was downregulated during the peak of viremia, suggesting impaired detoxification capacity and disruption of normal liver function. Collectively, these findings show that RVFV replication and pathogenesis in the liver depend on host-specific factors and lead to major dysregulation of signaling and metabolic pathways.

Recent advances in organoid and organotypic liver culture systems derived from both animal and human tissues could provide powerful platforms to investigate the molecular and metabolic determinants of RVFV replication and associated hepatic pathogenesis [55]. Along these lines, human liver spheroids have been shown to support RVFV infection, although relatively high viral inocula are required to achieve productive replication [56]. While this limitation may reduce their applicability for high-throughput antiviral screening, such models remain valuable for elucidating host cellular factors and metabolic pathways essential for RVFV replication and virus-host interactions. Identifying the metabolic routes that are essential for viral replication or contribute to disease progression will be crucial for pinpointing host-directed molecules capable of blocking viral replication and/or mitigating clinical manifestations.

### Neuropathogenesis and encephalitis

Encephalitis in humans is a severe and often fatal complication of RVFV infection, typically arising at later stages of disease. Neurological symptoms occur in 1–21% of cases, most often as delayed meningoencephalitis appearing 1–4 weeks post-infection, with mortality approaching 50%. Neurological involvement most often presents as encephalitis accompanied by dizziness, delirium, and sleep disturbance, and may progress to confusion, disorientation, or coma [57]. In some patients, RVFV encephalitis leads to severe neurological sequelae, including hemiparesis, a persistent vegetative state, and profound cognitive impairment. These complications may arise weeks to months after the acute phase and can become permanent, although their frequency and underlying mechanisms remain poorly documented [58]. In young ruminants (lambs and calves), RVFV infection typically presents as a rapidly fatal syndrome dominated by massive necrotizing hepatitis, with case fatality rates of 70–100% [59,60]. Neurological signs, including hyperexcitability, convulsions, encephalomyelitis, and coma, are more common in this age group than in adults.

In mice, viral antigens have been detected postmortem primarily in the hippocampus, cerebral cortex and, to a lesser extent, deep brain structures: regions showing large areas of necrotic neurons associated with lymphocytic infiltrates [7]. Similar observations have also been reported in aerosol-infected Lewis rats, with additional involvement of the olfactory bulb and meningeal layers [61]. Despite its clinical significance, the mechanisms underlying neuroinvasion and neuropathology remain poorly understood. Recent *in vivo* and *ex vivo* studies have begun to shed light on the early events of infection and the key features of RVFV neuropathogenesis. A growing body of evidence indicates that RVFV causes encephalitic disease primarily through direct viral injury to the CNS, while immune-mediated mechanisms, particularly microglial activation and cytokine-driven damage, may further contribute to disease progression at later stages [7]. Conversely, intrinsic antiviral responses mediated by mitochondrial antiviral-signaling protein (MAVS), a key adaptor in the RIG-I-like receptor pathway, can restrict CNS infection in intranasally infected mice [62]. This suggests that innate immune responses play a dual role, restricting viral spread while potentially exacerbating neuropathology. While most studies on RVFV neuropathogenesis have employed aerosol or intranasal exposure models, the pathways by which the virus gains access to the CNS under natural, vector-borne transmission conditions remain poorly understood.

In cases of peripheral infection, disease progression appears to depend on the efficiency of viral replication in the liver, which varies among strains, individuals, and species. In mice, when hepatic replication becomes massive and uncontrolled, fulminant hepatitis develops, often leading to rapid death. In contrast, when viral replication in the liver is limited or effectively contained (for instance, as observed in the absence of NSs or hepatic LRP1), infection may subsequently extend to other LRP1-expressing compartments, including the blood-brain barrier (BBB) and the nervous system [63–66], ultimately leading to a delayed neurological phase culminating in encephalitic disease in both mice and humans. Similar to Jamestown Canyon virus, another arbovirus from the order *Hareavirales* [67], RVFV may directly infect neurons either at peripheral sites or following hepatic dissemination.

Alternatively, although RVFV appears able to cross the BBB *in vitro* without causing disruption [68], a sublethal hepatic infection may nonetheless induce inflammation that transiently weakens the barrier and promotes neuroinvasion. Moreover, liver-derived viral populations might possess increased neurotropism and/or an enhanced ability to elicit inflammatory responses within the CNS, potentially due to selective pressures within the hepatic environment that favor certain variants or modulate the composition of viral particles, thereby influencing their infectivity and tissue tropism. Elucidating how liver-brain crosstalk and systemic inflammation may contribute to CNS invasion will therefore be critical for understanding RVFV neuropathogenesis under natural infection conditions. Notably, a newly established Collaborative Cross mouse line reliably develops encephalitis after peripheral inoculation, highlighting the influence of host genetics and offering a robust model to dissect viral and host determinants of neurovirulence [69].

In rats, aerosol infection enables the virus to access the CNS *via* the olfactory route, infecting the olfactory epithelium and spreading along neuronal axons [61]. Subsequent disease progression is characterized by extensive viral replication and a delayed disruption of the BBB, with increased vascular permeability occurring only at later stages, further indicating that RVFV neuroinvasion occurs independently of early BBB disruption [61,70]. Consistently, a human *in vitro* BBB model demonstrated that RVFV is able to cross the barrier in a strain-dependent but NSs-independent manner, while maintaining tight-junction integrity [68]. Once within the CNS, infection induces microglial activation and cytokine upregulation, with neutrophils and macrophages constituting the predominant infiltrating immune cell populations [71]. However, blocking neutrophil recruitment does not improve disease outcome, suggesting that inflammation is largely secondary to direct virus-induced damage. *Ex vivo* rat brain slice cultures further demonstrate that the pathogenic ZH501 strain replicates efficiently within neural tissue, infecting neurons and triggering microglial and astrocyte activation, apoptotic cell death, and a robust proinflammatory cytokine response [72]. These organotypic brain cultures therefore provide a physiologically relevant model to dissect host-virus interactions and neuroinflammatory processes.

Overall, animal models, brain organotypic cultures, and human BBB systems now offer robust and complementary platforms to investigate RVFV neuroinvasion, host-virus interactions, and strain-specific neurotropism. In addition to rodent models, a ferret model of RVFV infection has recently been described and reproduces key features of encephalitic disease, providing a useful system to study neurological manifestations and neuroinvasion mechanisms [73]. These models enable detailed exploration of neuron-astrocyte-microglia cross-talk, clarify the roles of host genetics and innate immunity in disease progression, and facilitate comparative analyses of neurotropic properties across diverse field isolates. Deciphering the viral and immune mechanisms that drive RVFV-associated neuropathology will be critical for the development of targeted interventions to prevent or treat encephalitic disease. To this end, experimental variables such as route of exposure, inoculation dose, and the use of low-passaged field strains must be carefully considered, as they profoundly influence disease trajectories and can otherwise obscure an accurate understanding of RVFV neuropathogenesis under natural conditions.

### Hemorrhagic fever

Beyond hepatitis and encephalitis, RVFV can also cause hemorrhagic fever in a small subset of patients, representing one of the most severe yet least frequent clinical outcomes. This form is characterized by fulminant hepatitis with massive hepatocellular necrosis, rapidly progressing to jaundice, coagulopathy, and multi-organ involvement [57]. Clinically, patients may also present with mucosal and gastrointestinal hemorrhages, epistaxis, hematemesis, and melena, often accompanied by hypotension, edema, and circulatory shock [11]. Despite its severe clinical presentation and high case fatality rate, our understanding of RVFV-associated hemorrhagic fever remains limited due to the scarcity of well-documented human cases and pathological material.

Fatal RVFV infections are characterized by profound cytokine dysregulation, with markedly elevated levels of IL-8, CXCL9, MCP-1, IP-10, and IL-10, accompanied by reduced levels of RANTES (CCL5) [74]. This skewed inflammatory profile is thought to promote endothelial damage, vascular inflammation, and disseminated coagulopathy, ultimately leading to capillary leakage and the hemorrhagic presentation typical of severe disease. In contrast, analysis of three surviving patients revealed that higher viral loads were associated with pronounced systemic inflammation evidenced by increased IP-10, CRP, Eotaxin, MCP-2, and Granzyme B together with elevated markers of fibrinolysis (tPA, D-dimer) and endothelial activation (sICAM-1) [75]. Notably, viral burden negatively correlated with P-selectin, ADAMTS13, and fibrinogen-related coagulation factors, suggesting that rising levels of these markers may signal gradual resolution of coagulopathy during recovery. Collectively, these findings indicate that hemorrhagic manifestations in RVF arise from complex interactions between viral replication, platelet activation, and dysregulated coagulation-fibrinolytic pathways, rather than from a single defective mechanism. Overall, current evidence indicates that severe hemorrhagic disease arises from the combined hepatocellular damage, impaired hemostasis, and inflammation-driven microvascular injury, although the relative contributions of viral and host factors remain unclear.

Deciphering the vascular processes leading to coagulopathy and endothelial damage may reveal therapeutic opportunities to mitigate RVFV-associated hemorrhagic disease. To this end, integrating *ex vivo* organotypic and cross-species comparative models will be crucial to define the underlying mechanisms and key determinants of vascular pathology. For example, RVFV infections in animal models highlight marked species-specific differences in the severity and outcome of hemorrhagic disease: in mice, RVFV typically induces acute hepatitis without hemorrhage [17], whereas Golden Syrian hamsters consistently develop fulminant, fatal hepatitis with extensive hemorrhagic lesions [76]. The mechanisms underlying these divergent phenotypes remain unclear, but one plausible explanation is that the hamster liver exhibits high susceptibility to RVFV, resulting in rapid hepatic failure associated with vascular damage. Comparative analyses of hepatic responses across species (mice, hamsters, and humans) could therefore elucidate key pathways governing species-specific susceptibility and hemorrhagic progression, including cytokine-mediated vascular dysfunction, and ultimately aid in identifying early biomarkers predictive of severe disease in humans.

## Placenta and retina: The overlooked tissues in Rift Valley fever pathogenesis

Reproductive complications of RVFV infection remain among the least studied aspects of pathogenesis, despite their major clinical and epidemiological importance [8]. In livestock, abortion storms cause devastating economic losses, with abortion rates reaching up to 100% among pregnant domestic ruminants. In humans, vertical transmission and adverse pregnancy outcomes have been reported but remain insufficiently documented [12].

### RVFV placental infection and vertical transmission

Experimental models have begun to clarify RVFV placental tropism. In late-gestation rats, the virus readily crosses the maternal-fetal interface, establishing infection in the decidua, basal zone, and labyrinth, where necrosis, hemorrhage, and inflammatory cytokine responses correlate with fetal demise [77]. Pregnancy outcomes range from normal live-born pups to fetuses exhibiting teratogenic abnormalities or undergoing intrauterine death. Although lesions occurred across all outcomes, teratogenic cases showed extensive multi-layer hemorrhage and strong induction of pro-inflammatory cytokines, type I IFNs, and chemokines, implicating inflammation as a driver of RVFV-associated teratogenicity [78]. Comparable findings have been reported in human and ruminant placentas. In human mid-gestation explants, RVFV infects chorionic villi, replicating in both the syncytiotrophoblast and underlying cytotrophoblasts [78,79]. In pregnant ewes, infection produces high placental viral loads, beginning in maternal epithelial cells and spreading to fetal trophoblasts. Notably, RVFV may exploit the haemophagous zone of ovine and human placentas, where direct contact between maternal blood and fetal trophoblasts facilitates transplacental transmission, a process that across species is associated with vascular injury, tissue necrosis, and inflammatory responses [79].

In sum, these findings demonstrate that RVFV efficiently targets the maternal-fetal interface and crosses the placental barrier through multiple routes, with inflammation-driven tissue damage likely underpinning adverse pregnancy outcomes in both experimental models and natural infections. Human syncytiotrophoblasts are usually resistant to viral infection because they secrete antiviral type III IFNs [80,81]. However, both type I and type III IFNs rely on the JAK/STAT pathway which is actively antagonized by the RVFV NSs protein [82]. This suggests that NSs may enhance viral replication in placental tissues by downregulating JAK/STAT signaling, even though RVFV can cross the placental barrier in the absence of NSs in ovine models, as shown for the attenuated Clone 13 strain [83]. Even so, the viral strategies that circumvent this barrier, the impact of gestational stage on placental susceptibility, and the role of fetal immune responses remain poorly defined and should be prioritized in future studies to clarify the mechanisms of RVFV vertical transmission and pathogenesis. Moreover, LRP1 is abundantly expressed in human placental tissue [84,85], where it may contribute to lipid transport [86]. However, its potential involvement in RVFV placental infection remains to be elucidated. Finally, understanding placental infection is also crucial for vaccine safety assessment, as illustrated by the previously mentioned Clone 13 data [83].

### RVFV ocular disease and pathogenesis

Ocular disease, typically manifesting as retinitis, has been reported in humans and affects approximately 2–5% of all human RVFV infections, rising to up to 10% in severe cases [2]. Symptoms typically resolved within two weeks, but in some cases persisted for three months or more [57]. In severe forms, retinal scarring can cause permanent vision impairment, with blindness occurring in nearly half of affected patients. Retinal lesions are thus a major cause of irreversible vision loss, with up to 71% of affected eyes progressing to blindness [58]. In human RVFV infection, ocular involvement often presents as bilateral retinal lesions with associated hemorrhage and edema, while uveitis and optic nerve or vascular complications are observed in a subset of cases [87–89]. Despite the clinical importance of this outcome, the mechanisms of RVFV ocular pathogenesis remain poorly understood.

Experimental infections in Sprague Dawley rats have shown that subcutaneous inoculation leads to viral dissemination to the uvea, retina, and optic nerve, accompanied by posterior eye inflammation and cytokine induction [90]. Yet, the precise cellular targets of RVFV infection within the eye and the mechanisms by which the virus crosses the blood-ocular barrier remain unresolved. Future research should aim to elucidate these processes to improve our understanding of ocular disease and develop strategies for its prevention and treatment. Several *ex vivo* and *in vitro* human retinal models, such as tissue explants, retino-spheres, and stem cell-derived organoids, have been developed to study retinal development and disease [91]. These models could prove valuable for better understanding RVFV infection and the associated retinal pathology.

Altogether, placental and ocular tissues are both susceptible to RVFV infection and may underlie severe or persistent disease outcomes. Despite recent progress, particularly from rodent studies, these manifestations, most notably ocular disease, remain poorly characterized and merit focused investigation. Whether RVFV infection of ocular tissues is linked to CNS involvement remains uncertain, particularly given the direct neuronal pathway provided by the optic nerve. Integrating placental and ocular models with animal studies will deepen our understanding of tissue-specific infection mechanisms and may reveal therapeutic opportunities to protect vulnerable compartments such as the maternal-fetal interface and sensitive organs including the eye.

## Conclusion and priority research agenda

RVFV does not induce uniform disease but rather produces a spectrum of clinical manifestations depending on the infection routes, strain-specific viral genetic determinants, host responses, and organ-specific replication dynamics. Its segmented genome allows reassortment, generating unpredictable phenotypes that challenge surveillance and risk assessment. Likewise, differences in exposure route, viral determinants, and host species, as well as the age and immunological status of the infected host, shape whether disease manifests as fulminant hepatitis, delayed neuroinvasion, or other organ-specific outcomes. This complexity underscores the need to move beyond simplified laboratory models and toward integrated experimental systems that better reflect natural transmission routes, incorporate the diversity of circulating field isolates, and leverage advanced *ex vivo* platforms such as organoids and organotypic cultures.

Deep sequencing of circulating strains, combined with reverse-genetic approaches, will be critical to identifying virulence determinants, monitoring the emergence of novel variants, and resolving the intra-strain genetic diversity that shapes clinical outcomes. Systematic characterization of circulating isolates using standardized protocols will be required to dissect the molecular basis of hepatic *versus* neural disease and to anticipate the evolutionary consequences of reassortment. Underexplored manifestations, such as placental infection and abortion in ruminants (and possibly humans), as well as retinitis and vision loss in human cases, should likewise be incorporated into this framework. Adopting an organ-centered perspective is therefore essential for refining experimental strategies, explaining species- and strain-specific differences, and identifying the viral and host factors that drive the diverse clinical manifestations of Rift Valley fever.

**Figure 2.**
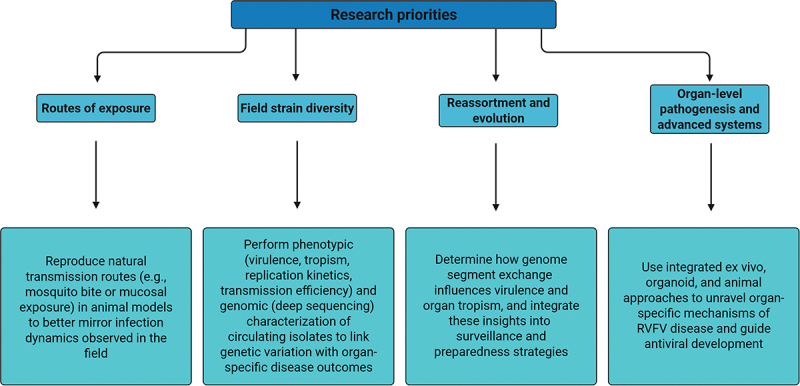
Strategic research priorities to advance understanding of Rift Valley fever virus (RVFV) pathogenesis. Created with BioRender.com.

A more integrated exploration of viral, host, and environmental factors, particularly the mosquito vector, which represents both a natural infection route and an ecological hub for reassortment, will be equally critical to unravel the complex mechanisms underlying RVFV pathogenesis. With climate change and ongoing outbreaks, such as the recent episode in northern Senegal and Mauritania in September 2025 involving persistent lineage H (as per the nomenclature of Grobbelaar *et al*. [92]), marked by several hemorrhagic cases and increased human mortality [93], bridging these knowledge gaps is not only an academic challenge but an urgent global health priority. Recent international roadmaps emphasize the need to deepen our understanding of RVFV ecology, transmission dynamics, and One Health surveillance in endemic and hyperendemic settings [94]. Complementing these ecological and epidemiological perspectives, we outline four research priorities aimed at elucidating the mechanistic basis of RVFV pathogenesis and guiding the development of robust experimental frameworks for future investigations (Figure 2).

## Data Availability

There is no data associated with this research.

## References

[1] Ikegami T. Molecular biology and genetic diversity of Rift Valley fever virus. Antiviral Res. 2012;95(3):293–14. doi: 10.1016/j.antiviral.2012.06.001 22710362 PMC3586937

[2] Wright D, Kortekaas J, Bowden TA, et al. Rift Valley fever: biology and epidemiology. J Gen Virol. 2019;100(8):1187–1199. doi: 10.1099/jgv.0.001296 31310198 PMC7613496

[3] Pepin M, Bouloy M, Bird BH, et al. Rift Valley fever virus (Bunyaviridae: Phlebovirus): an update on pathogenesis, molecular epidemiology, vectors, diagnostics and prevention. Vet Res. 2010;41(6):61. doi: 10.1051/vetres/2010033 21188836 PMC2896810

[4] Martin V, Chevalier V, Ceccato P, et al. The impact of climate change on the epidemiology and control of Rift Valley fever. Rev Sci Tech. 2008;27(2):413–426. 18819669

[5] Gibson S, Noronha LE, Tubbs H, et al. The increasing threat of Rift Valley fever virus globalization: strategic guidance for protection and preparation. J Med Entomol. 2023;60(6):1197–1213. doi: 10.1093/jme/tjad113 37862067

[6] Duboeuf M, Legrand AF, Lozach PY. NSs: the multifaceted bunyavirus virulence factor. Npj Viruses. 2025;3(1):65. doi: 10.1038/s44298-025-00146-5 40903519 PMC12408830

[7] Wilson LR, McElroy AK. Rift Valley fever virus encephalitis: viral and host determinants of pathogenesis. Annu Rev Virol. 2024;11(1):309–325. doi: 10.1146/annurev-virology-093022-011544 38635867 PMC11427164

[8] O’Neill L, Gubbins S, Reynolds C, et al. The socioeconomic impacts of Rift Valley fever: a rapid review. PLOS Negl Trop Dis. 2024;18(8):e0012347. doi: 10.1371/journal.pntd.0012347 39207938 PMC11361445

[9] Xu Y, Wang X, Jiang L, et al. Natural hosts and animal models for Rift Valley fever phlebovirus. Front Vet Sci. 2023;10:1258172. doi: 10.3389/fvets.2023.1258172 37929288 PMC10621046

[10] Nair N, Osterhaus A, Rimmelzwaan GF, et al. Rift Valley fever virus-infection, pathogenesis and host immune responses. Pathogens. 2023;12(9):1174. doi: 10.3390/pathogens12091174 37764982 PMC10535968

[11] Odendaal L, Davis AS, Venter EH. Insights into the pathogenesis of viral haemorrhagic fever based on virus tropism and tissue lesions of natural Rift Valley fever. Viruses. 2021;13(4):709. doi: 10.3390/v13040709 33923863 PMC8073615

[12] McMillen CM, Hartman AL. Rift Valley fever: a threat to pregnant women hiding in plain sight? J Virol. 2021;95(9). doi: 10.1128/JVI.01394-19 PMC8104100 33597209

[13] Odendaal L, Clift SJ, Fosgate GT, et al. Lesions and cellular tropism of natural Rift Valley fever virus infection in adult sheep. Vet Pathol. 2019;56(1):61–77. doi: 10.1177/0300985818806049 30343650

[14] Grossi-Soyster EN, Lee J, King CH, et al. The influence of raw milk exposures on Rift Valley fever virus transmission. PLOS Negl Trop Dis. 2019;13(3):e0007258. doi: 10.1371/journal.pntd.0007258 30893298 PMC6443189

[15] Cook EAJ, Grossi-Soyster EN, de Glanville WA, et al. The sero-epidemiology of Rift Valley fever in people in the Lake Victoria Basin of western Kenya. PLOS Negl Trop Dis. 2017;11(7):e0005731. doi: 10.1371/journal.pntd.0005731 28686589 PMC5517073

[16] Lumley S, Horton DL, Hernandez-Triana LLM, et al. Rift Valley fever virus: strategies for maintenance, survival and vertical transmission in mosquitoes. J Gen Virol. 2017;98(5):875–887. doi: 10.1099/jgv.0.000765 28555542

[17] Smith DR, Steele KE, Shamblin J, et al. The pathogenesis of Rift Valley fever virus in the mouse model. Virology. 2010;407(2):256–267. doi: 10.1016/j.virol.2010.08.016 20850165

[18] Kroeker AL, Smid V, Embury-Hyatt C, et al. Increased susceptibility of cattle to intranasal RVFV infection. Front Vet Sci. 2020;7:137. doi: 10.3389/fvets.2020.00137 32411730 PMC7200984

[19] Le Coupanec A, Babin D, Fiette L, et al. Aedes mosquito saliva modulates Rift Valley fever virus pathogenicity. PLOS Negl Trop Dis. 2013;7(6):e2237. doi: 10.1371/journal.pntd.0002237 23785528 PMC3681724

[20] Reed C, Lin K, Wilhelmsen C, et al. Aerosol exposure to Rift Valley fever virus causes earlier and more severe neuropathology in the murine model, which has important implications for therapeutic development. PLOS Negl Trop Dis. 2013;7(4):e2156. doi: 10.1371/journal.pntd.0002156 23593523 PMC3617210

[21] Lacote S, Tamietti C, Chabert M, et al. Intranasal exposure to Rift Valley fever virus live-attenuated strains leads to high mortality rate in immunocompetent mice. Viruses. 2022;14(11):2470. doi: 10.3390/v14112470 36366567 PMC9694885

[22] Graham VA, Easterbrook L, Kennedy E, et al. Pathogenesis of Rift Valley fever virus in a BALB/c mouse model is affected by virus culture conditions and sex of the animals. Viruses. 2023;15(12):2369. doi: 10.3390/v15122369 38140610 PMC10747589

[23] Bird BH, Khristova ML, Rollin PE, et al. Complete genome analysis of 33 ecologically and biologically diverse Rift Valley fever virus strains reveals widespread virus movement and low genetic diversity due to recent common ancestry. J Virol. 2007;81(6):2805–2816. doi: 10.1128/JVI.02095-06 17192303 PMC1865992

[24] Battles JK, Dalrymple JM. Genetic variation among geographic isolates of Rift Valley fever virus. Am J Trop Med Hyg. 1988;39(6):617–631. doi: 10.4269/ajtmh.1988.39.617 2462795

[25] Anderson GW, Peters CJ. Viral determinants of virulence for Rift Valley fever (RVF) in rats. Microb Pathog. 1988;5(4):241–250. doi: 10.1016/0882-4010(88)90096-4 3266284

[26] Ikegami T, Balogh A, Nishiyama S, et al. Distinct virulence of Rift Valley fever phlebovirus strains from different genetic lineages in a mouse model. PLOS ONE. 2017;12(12):e0189250. doi: 10.1371/journal.pone.0189250 29267298 PMC5739399

[27] Chabert M, Lacote S, Marianneau P, et al. Comparative study of two Rift Valley fever virus field strains originating from Mauritania. PLOS Negl Trop Dis. 2024;18(12):e0012728. doi: 10.1371/journal.pntd.0012728 39652604 PMC11658707

[28] Kuhn JH, Brown K, Adkins S, et al. Promotion of order bunyavirales to class Bunyaviricetes to accommodate a rapidly increasing number of related polyploviricotine viruses. J Virol. 2024;98(10):e0106924. doi: 10.1128/jvi.01069-24 39303014 PMC11494962

[29] Gerrard SR, Li L, Barrett AD, et al. Ngari virus is a Bunyamwera virus reassortant that can be associated with large outbreaks of hemorrhagic fever in Africa. J Virol. 2004;78(16):8922–8926. doi: 10.1128/JVI.78.16.8922-8926.2004 15280501 PMC479050

[30] Sall AA, Zanotto PM, Sene OK, et al. Genetic reassortment of Rift Valley fever virus in nature. J Virol. 1999;73(10):8196–8200. doi: 10.1128/JVI.73.10.8196-8200.1999 10482570 PMC112837

[31] Harris EK, Balaraman V, Keating CC, et al. Co-infection of Culex tarsalis mosquitoes with Rift Valley fever phlebovirus strains results in efficient viral reassortment. Viruses. 2025;17(1):88. doi: 10.3390/v17010088 39861876 PMC11768849

[32] Balaraman V, Indran SV, Kim IJ, et al. Rift Valley fever phlebovirus reassortment study in sheep. Viruses. 2024;16(6):880. doi: 10.3390/v16060880 38932172 PMC11209395

[33] Gaudreault NN, Indran SV, Balaraman V, et al. Molecular aspects of Rift Valley fever virus and the emergence of reassortants. Virus Genes. 2019;55(1):1–11. doi: 10.1007/s11262-018-1611-y 30426314

[34] Koch J, Xin Q, Tischler ND, et al. Entry of phenuiviruses into mammalian host cells. Viruses. 2021;13(2):299. doi: 10.3390/v13020299 33672975 PMC7918600

[35] Vialat P, Billecocq A, Kohl A, et al. The S segment of Rift Valley fever phlebovirus (Bunyaviridae) carries determinants for attenuation and virulence in mice. J Virol. 2000;74(3):1538–1543. doi: 10.1128/JVI.74.3.1538-1543.2000 10627566 PMC111490

[36] Ly HJ, Ikegami T. Rift Valley fever virus NSs protein functions and the similarity to other bunyavirus NSs proteins. Virol J. 2016;13(1):118. doi: 10.1186/s12985-016-0573-8 27368371 PMC4930582

[37] Petraccione K, Omichinski JG, Kehn-Hall K. Immune evasion by the NSs protein of Rift Valley fever virus: a viral houdini act. Viruses. 2025;17(10):1398. doi: 10.3390/v17101398 41157666 PMC12567723

[38] Swanepoel R, Blackburn NK. Demonstration of nuclear immunofluorescence in Rift Valley fever infected cells. J Gen Virol. 1977;34(3):557–561. doi: 10.1099/0022-1317-34-3-557 323417

[39] Leger P, Nachman E, Richter K, et al. NSs amyloid formation is associated with the virulence of Rift Valley fever virus in mice. Nat Commun. 2020;11(1):3281. doi: 10.1038/s41467-020-17101-y 32612175 PMC7329897

[40] Li H, Zhang Y, Rao G, et al. Rift valley fever virus coordinates the assembly of a programmable E3 ligase to promote viral replication. Cell. 2024;187(24):6896–6913.e15. doi: 10.1016/j.cell.2024.09.008 39366381

[41] Dodd KA, McElroy AK, Jones TL, et al. Rift valley fever virus encephalitis is associated with an ineffective systemic immune response and activated T cell infiltration into the CNS in an immunocompetent mouse model. PLOS Negl Trop Dis. 2014;8(6):e2874. doi: 10.1371/journal.pntd.0002874 24922480 PMC4055548

[42] Bird BH, Albarino CG, Nichol ST. Rift Valley fever virus lacking NSm proteins retains high virulence in vivo and may provide a model of human delayed onset neurologic disease. Virology. 2007;362(1):10–15. doi: 10.1016/j.virol.2007.01.046 17412386

[43] Kreher F, Tamietti C, Gommet C, et al. The Rift Valley fever accessory proteins NSm and P78/NSm-GN are distinct determinants of virus propagation in vertebrate and invertebrate hosts. Emerg Microbes Infect. 2014;3(10):e71. 26038497 10.1038/emi.2014.71 PMC4217093

[44] Terasaki K, Won S, Makino S. The C-terminal region of Rift Valley fever virus NSm protein targets the protein to the mitochondrial outer membrane and exerts antiapoptotic function. J Virol. 2013;87(1):676–682. doi: 10.1128/JVI.02192-12 23097454 PMC3536385

[45] Won S, Ikegami T, Peters CJ, et al. NSm protein of Rift Valley fever virus suppresses virus-induced apoptosis. J Virol. 2007;81(24):13335–13345. doi: 10.1128/JVI.01238-07 17913816 PMC2168885

[46] Terasaki K, Kalveram B, Johnson KN, et al. Rift Valley fever virus 78kDa envelope protein attenuates virus replication in macrophage-derived cell lines and viral virulence in mice. PLOS Negl Trop Dis. 2021;15(9):e0009785. doi: 10.1371/journal.pntd.0009785 34516560 PMC8460012

[47] Léger P, Tetard M, Youness B, et al. Differential use of the C-type lectins L-SIGN and DC-SIGN for phlebovirus endocytosis. Traffic. 2016;17(6):639–656. doi: 10.1111/tra.12393 26990254

[48] Pietrantoni A, Fortuna C, Remoli ME, et al. Bovine lactoferrin inhibits Toscana virus infection by binding to heparan sulphate. Viruses. 2015;7(2):480–495. doi: 10.3390/v7020480 25643293 PMC4353899

[49] de Boer SM, Kortekaas J, de Haan CA, et al. Heparan sulfate facilitates Rift Valley fever virus entry into the cell. J Virol. 2012;86(24):13767–13771. doi: 10.1128/JVI.01364-12 23015725 PMC3503077

[50] Lozach PY, Kuhbacher A, Meier R, et al. DC-SIGN as a receptor for phleboviruses. Cell Host Microbe. 2011;10(1):75–88. doi: 10.1016/j.chom.2011.06.007 21767814

[51] Ganaie SS, Schwarz MM, McMillen CM, et al. Lrp1 is a host entry factor for Rift Valley fever virus. Cell. 2021;184(20):5163–5178 e24. doi: 10.1016/j.cell.2021.09.001 34559985 PMC8786218

[52] Schwarz MM, Ganaie SS, Feng A, et al. Lrp1 is essential for lethal Rift Valley fever hepatic disease in mice. Sci Adv. 2023;9(28):eadh2264. doi: 10.1126/sciadv.adh2264 37450601 PMC10348670

[53] Devignot S, Sha TW, Burkard TR, et al. Low-density lipoprotein receptor-related protein 1 (LRP1) as an auxiliary host factor for RNA viruses. Life Sci Alliance. 2023;6(7):e202302005. doi: 10.26508/lsa.202302005 37072184 PMC10114362

[54] Bermudez-Mendez E, Angelino P, van Keulen L, et al. Transcriptomic profiling reveals intense host-pathogen dispute compromising homeostasis during acute Rift Valley fever virus infection. J Virol. 2023;97(6):e0041523. doi: 10.1128/jvi.00415-23 37306574 PMC10308945

[55] Luce E, Messina A, Duclos-Vallee JC. Hepatic organoids as a platform for liver disease modeling and the development of novel therapies. Clin Res Hepatol Gastroenterol. 2025;49(7):102647. doi: 10.1016/j.clinre.2025.102647 40615111

[56] Chaput S, Driouich JS, Gruber S, et al. Assessing human liver spheroids as a model for antiviral drug evaluation against BSL-3 haemorrhagic fever viruses. Antiviral Res. 2025;239:106188. doi: 10.1016/j.antiviral.2025.106188 40360123

[57] Anywaine Z, Lule SA, Hansen C, et al. Clinical manifestations of Rift Valley fever in humans: systematic review and meta-analysis. PLOS Negl Trop Dis. 2022;16(3):e0010233. doi: 10.1371/journal.pntd.0010233 35333856 PMC8986116

[58] Javelle E, Lesueur A, Pommier de Santi V, et al. The challenging management of Rift Valley fever in humans: literature review of the clinical disease and algorithm proposal. Ann Clin Microbiol Antimicrob. 2020;19(1):4. doi: 10.1186/s12941-020-0346-5 31969141 PMC6977312

[59] Coetzer JA. The pathology of Rift Valley fever. I. Lesions occurring in natural cases in new-born lambs. Onderstepoort J Vet Res. 1977;44(4):205–211. 613292

[60] Rippy MK, Topper MJ, Mebus CA, et al. Rift valley fever virus-induced encephalomyelitis and hepatitis in calves. Vet Pathol. 1992;29(6):495–502. doi: 10.1177/030098589202900602 1448895

[61] Boyles DA, Schwarz MM, Albe JR, et al. Development of rift valley fever encephalitis in rats is mediated by early infection of olfactory epithelium and neuroinvasion across the cribriform plate. J Gen Virol. 2021;102(2). doi: 10.1099/jgv.0.001522 PMC8116942 33231535

[62] Hum NR, Bourguet FA, Sebastian A, et al. MAVS mediates a protective immune response in the brain to Rift Valley fever virus. PLOS Pathog. 2022;18(5):e1010231. doi: 10.1371/journal.ppat.1010231 35584192 PMC9154093

[63] Auderset L, Cullen CL, Young KM. Low density lipoprotein-receptor related protein 1 is differentially expressed by neuronal and glial populations in the developing and mature mouse central nervous system. PLOS ONE. 2016;11(6):e0155878. doi: 10.1371/journal.pone.0155878 27280679 PMC4900551

[64] Faissner A. Low-density lipoprotein receptor-related protein-1 (LRP1) in the glial lineage modulates neuronal excitability. Front Netw Physiol. 2023;3:1190240. doi: 10.3389/fnetp.2023.1190240 37383546 PMC10293750

[65] Liu Q, Zhang J, Tran H, et al. Lrp1 shedding in human brain: roles of ADAM10 and ADAM17. Mol Neurodegener. 2009;4(1):17. doi: 10.1186/1750-1326-4-17 19371428 PMC2672942

[66] Ma Q, Zhao Z, Sagare AP, et al. Blood-brain barrier-associated pericytes internalize and clear aggregated amyloid-beta42 by LRP1-dependent apolipoprotein E isoform-specific mechanism. Mol Neurodegener. 2018;13(1):57. 30340601 10.1186/s13024-018-0286-0 PMC6194676

[67] Frey ZD, Price DA, Connors KA, et al. Lrp1 facilitates Jamestown Canyon virus infection of neurons. J Virol. 2025;99(12):e0184125. doi: 10.1128/jvi.01841-25 41313001 PMC12724265

[68] Quellec J, Piro-Megy C, Cannac M, et al. Rift Valley fever virus is able to cross the human blood-brain barrier in vitro by direct infection with no deleterious effects. J Virol. 2024;98(10):e0126724. doi: 10.1128/jvi.01267-24 39345143 PMC11494904

[69] Cartwright HN, Barbeau DJ, Doyle JD, et al. Genetic diversity of Collaborative Cross mice enables identification of novel Rift Valley fever virus encephalitis model. PLOS Pathog. 2022;18(7):e1010649. doi: 10.1371/journal.ppat.1010649 35834486 PMC9282606

[70] Walters AW, Kujawa MR, Albe JR, et al. Vascular permeability in the brain is a late pathogenic event during Rift Valley fever virus encephalitis in rats. Virology. 2019;526:173–179. doi: 10.1016/j.virol.2018.10.021 30396029 PMC6286220

[71] Albe JR, Boyles DA, Walters AW, et al. Neutrophil and macrophage influx into the central nervous system are inflammatory components of lethal Rift Valley fever encephalitis in rats. PLOS Pathog. 2019;15(6):e1007833. doi: 10.1371/journal.ppat.1007833 31220182 PMC6605717

[72] Connors KA, Frey ZD, Demers MJ, et al. Acute Rift Valley fever virus infection induces inflammatory cytokines and cell death in ex vivo rat brain slice culture. J Gen Virol. 2024;105(3). doi: 10.1099/jgv.0.001970 PMC10995633 38546100

[73] Barbeau DJ, Albe JR, Nambulli S, et al. Rift Valley fever virus infection causes acute encephalitis in the ferret. mSphere. 2020;5(5). doi: 10.1128/mSphere.00798-20 PMC7593599 33115835

[74] Jansen van Vuren P, Shalekoff S, Grobbelaar AA, et al. Serum levels of inflammatory cytokines in Rift Valley fever patients are indicative of severe disease. Virol J. 2015;12(1):159. doi: 10.1186/s12985-015-0392-3 26437779 PMC4595326

[75] de St Maurice A, Harmon J, Nyakarahuka L, et al. Rift valley fever viral load correlates with the human inflammatory response and coagulation pathway abnormalities in humans with hemorrhagic manifestations. PLOS Negl Trop Dis. 2018;12(5):e0006460. doi: 10.1371/journal.pntd.0006460 29727450 PMC5955566

[76] Scharton D, Van Wettere AJ, Bailey KW, et al. Rift Valley fever virus infection in golden Syrian hamsters. PLOS ONE. 2015;10(1):e0116722. doi: 10.1371/journal.pone.0116722 25607955 PMC4301868

[77] McMillen CM, Boyles DA, Kostadinov SG, et al. Congenital Rift Valley fever in Sprague Dawley rats is associated with diffuse infection and pathology of the placenta. PLOS Negl Trop Dis. 2022;16(10):e0010898. doi: 10.1371/journal.pntd.0010898 36315601 PMC9648853

[78] McMillen CM, Arora N, Boyles DA, et al. Rift valley fever virus induces fetal demise in Sprague-Dawley rats through direct placental infection. Sci Adv. 2018;4(12):eaau9812. doi: 10.1126/sciadv.aau9812 30525107 PMC6281433

[79] Oymans J, Wichgers Schreur PJ, van Keulen L, et al. Rift Valley fever virus targets the maternal-foetal interface in ovine and human placentas. PLOS Negl Trop Dis. 2020;14(1):e0007898. doi: 10.1371/journal.pntd.0007898 31961862 PMC6994196

[80] Bayer A, Lennemann NJ, Ouyang Y, et al. Type III interferons produced by human placental trophoblasts confer protection against Zika virus infection. Cell Host Microbe. 2016;19(5):705–712. doi: 10.1016/j.chom.2016.03.008 27066743 PMC4866896

[81] Wells AI, Coyne CB. Type III interferons in antiviral defenses at barrier surfaces. Trends Immunol. 2018;39(10):848–858. doi: 10.1016/j.it.2018.08.008 30219309 PMC6179363

[82] Benferhat R, Josse T, Albaud B, et al. Large-scale chromatin immunoprecipitation with promoter sequence microarray analysis of the interaction of the NSs protein of Rift Valley fever virus with regulatory DNA regions of the host genome. J Virol. 2012;86(20):11333–11344. doi: 10.1128/JVI.01549-12 22896612 PMC3457170

[83] Makoschey B, van Kilsdonk E, Hubers WR, et al. Rift Valley fever vaccine virus clone 13 is able to cross the ovine placental barrier associated with foetal infections, malformations, and stillbirths. PLOS Negl Trop Dis. 2016;10(3):e0004550. doi: 10.1371/journal.pntd.0004550 27031621 PMC4816553

[84] Jensen PH, Moestrup SK, Gliemann J. Purification of the human placental alpha 2-macroglobulin receptor. FEBS Lett. 1989;255(2):275–280. 2477279 10.1016/0014-5793(89)81105-6

[85] Jensen PH, Moestrup SK, Sottrup-Jensen L, et al. Receptors for alpha 2-macroglobulin- and pregnancy zone protein-proteinase complexes in the human placental syncytiotrophoblast. Placenta. 1988;9(5):463–477. doi: 10.1016/0143-4004(88)90019-7 2464820

[86] Gafvels ME, Coukos G, Sayegh R, et al. Regulated expression of the trophoblast alpha 2-macroglobulin receptor/low density lipoprotein receptor-related protein. Differentiation and cAMP modulate protein and mRNA levels. J Biol Chem. 1992;267(29):21230–21234. doi: 10.1016/S0021-9258(19)36822-X 1328226

[87] Al-Hazmi A, Al-Rajhi AA, Abboud EB, et al. Ocular complications of Rift Valley fever outbreak in Saudi Arabia. Ophthalmology. 2005;112(2):313–318. doi: 10.1016/j.ophtha.2004.09.018 15691569

[88] Freed I. Rift valley fever in man, complicated by retinal changes and loss of vision. S Afr Med J. 1951;25(50):930–932. 14892953

[89] Siam AL, Meegan JM, Gharbawi KF. Rift Valley fever ocular manifestations: observations during the 1977 epidemic in Egypt. Br J Ophthalmol. 1980;64(5):366–374. doi: 10.1136/bjo.64.5.366 7192158 PMC1043698

[90] Schwarz MM, Connors KA, Davoli KA, et al. Rift Valley fever virus infects the posterior segment of the eye and induces inflammation in a rat model of ocular disease. J Virol. 2022;96(20):e0111222. doi: 10.1128/jvi.01112-22 36194021 PMC9599513

[91] Eldred KC, Reh TA. Human retinal model systems: strengths, weaknesses, and future directions. Dev Biol. 2021;480:114–122. doi: 10.1016/j.ydbio.2021.09.001 34529997

[92] Grobbelaar AA, Weyer J, Leman PA, et al. Molecular epidemiology of Rift Valley fever virus. Emerg Infect Dis. 2011;17(12):2270–2276. doi: 10.3201/eid1712.111035 22172568 PMC3311189

[93] Diagne MM, Fall G, Sall A, et al. Molecular characterization of Rift Valley fever virus from the 2025 outbreak in northern Senegal reveals lineage H persistence and key polymerase mutations. J Med Virol. 2025;97(12):e70734. doi: 10.1002/jmv.70734 41319301 PMC12665341

[94] Rostal MK, Thompson PN, Anyamba A, et al. Rift Valley fever epidemiology: shifting the paradigm and rethinking research priorities. Lancet Planet Health. 2025;9(9):101299. doi: 10.1016/j.lanplh.2025.101299 40921175

